# Suddenly Telework: Job Crafting as a Way to Promote Employee Well-Being?

**DOI:** 10.3389/fpsyg.2021.790862

**Published:** 2022-01-14

**Authors:** Christiane R. Stempel, Katja Siestrup

**Affiliations:** Department of Work and Organizational Psychology, Faculty of Psychology, FernUniversität Hagen, Hagen, Germany

**Keywords:** pandemic (COVID-19), working conditions, job crafting, telework, mix-method approach, well-being

## Abstract

COVID-19 confronted many people with an abrupt shift from their usual working environment to telework. This study explores which job characteristics are perceived as most crucial in this exceptional situation and how they differ from people’s previous working conditions. Additionally, we focus on job crafting as a response to this situation and how it is related to employees’ well-being. We conducted an online survey with *N* = 599 participants, of which 321 reported that they were telework newcomers. First, we asked participants to indicate the three most important advantages and disadvantages they see in telework. The subsequent questionnaire contained a comprehensive measure of working conditions before and during the pandemic, job crafting behaviors, and indicators of well-being. Based on the qualitative answers, we identified three major advantages and disadvantages. Quantitative results indicate perceived changes in all job characteristics for telework newcomers. Concerning working conditions and well-being, job crafting activities that aim to increase structural and social resources are important mediators. The findings underline the need to design appropriate telework conditions and encourage job crafting activities to foster occupational well-being.

## Introduction

The COVID-19 pandemic brought massive changes for telework, because the established regulations encouraged many people to work from home (Baert et al., 2020). The suddenness of the change might be challenging, as time to adjust to the new working conditions and communication processes is considered important for successful telework (Maruyama and Tietze, 2012). Since the pandemic constitutes a significant additional stressor in people’s lives, it seems particularly important to examine its impact on employee well-being and health (Rudolph et al., 2020; Meyer et al., 2021). Exploring the involuntary transition to work at home provides an opportunity to investigate the most important working conditions in the given situation, examine how people adjust to them and how this in turn relates to their well-being. Regarding the adjustment, it seems worthwhile to take a closer look at job crafting, because these proactive behaviors that aim at improving the work environment (Tims and Bakker, 2010) could be important in facilitating transition processes (Petrou et al., 2017; Gascoigne and Kelliher, 2018).

Past research clearly shows that working conditions are crucial for employees’ well-being and health in regular and teleworking settings (e.g., Sardeshmukh et al., 2012; Barber and Santuzzi, 2015; Nielsen et al., 2017). One way to actively shape one’s work environment and thus deal with an exceptional situation imposed by the COVID-19 regulations is to engage in job crafting activities (Tims and Bakker, 2010; Dettmers and Mülder, 2020). Thus, we draw on the job demands-resources model (JD-R, Demerouti et al., 2001) to identify central job conditions by asking participants about their perceptions of the three most important advantages and disadvantages. Based on the JD-R model, we assume that perceived advantages are seen as resources (or reduced demands) and are associated with more work engagement and less emotional exhaustion. In contrast, disadvantages should show the opposite relationships (Bakker and Demerouti, 2017). Furthermore, we assume that job crafting behaviors play an important role, as they might indicate how people adjust to the situation in terms of creating a healthy work environment (Lichtenthaler and Fischbach, 2018; Boehnlein and Baum, 2020). Job crafting is a particular form of proactive behavior which employees use to improve job characteristics instead of waiting for employers’ action (Demerouti, 2014). This might be especially relevant for teleworking in the pandemic situation, because employers might have limited possibilities to create individualized job characteristics. We expect job resources and demands to be related to job crafting activities, the first as enablers and the second as reasons. In turn, job crafting activities should be associated with improved work engagement and less emotional exhaustion (Tims et al., 2013; Bakker and Demerouti, 2017). To further explore the role of experience with telework situations (Maruyama and Tietze, 2012), we aim to compare newcomers to the situation with people working from home before the pandemic.

We contribute to the current literature on teleworking conditions by investigating how an abrupt change caused by the pandemic has influenced employees’ perceptions of their working conditions. Moreover, we apply a qualitative approach to identify and weigh the working conditions that are considered most central in that exceptional context and combine the qualitative with quantitative data in our mixed-method approach. This way, we additionally compare how experienced teleworkers assess the working conditions at home compared to newcomers. Finally, by means of a mediation, we take a closer look at employees’ job crafting activities as a strategy to shape their working conditions and improve their well-being. Addressing these questions could help provide valuable insights into which working conditions need to be prioritized in the telework context and which job crafting behaviors could facilitate a transition.

## Theory

### Working Conditions During the Pandemic

To investigate job demands and resources during the pandemic, we draw on the JD-R model (Demerouti et al., 2001). According to the JD-R model, job demands include “physical, psychological, social, or organizational aspects of the job that require sustained physical and/or psychological (cognitive and emotional) effort or skills” (Bakker and Demerouti, 2007, p. 312). The model postulates that extensive job demands strain individuals over time, resulting in detrimental personal, group, or organizational outcomes (Bakker and Demerouti, 2017). Contrarily, job resources are the aspects of work that enable individuals to deal with job demands, motivate them, and stimulate personal development (Bakker et al., 2005; Bakker and Demerouti, 2007). Therefore, job resources unfold motivation, which in turn is associated with beneficial outcomes (Bakker and Demerouti, 2007; Hakanen et al., 2008). A variety of cross-sectional and longitudinal research supports the assumed pathways related to well-being and health conceptualized in the JD-R model (e.g., Bakker et al., 2005; Schaufeli and Taris, 2014; Bakker and Demerouti, 2017).

Due to the sudden onset of the pandemic and the need to work from home, many people’s working conditions have changed abruptly (Baert et al., 2020). On the one hand, adapting to the new working situation can be seen as an additional contextual demand, likely causing psychological strain as proposed by the JD-R model (Demerouti et al., 2001). For instance, people have to get used to not talking directly to their colleagues but using new modes of communication like digital conferences and messenger services instead (Nakrošienė et al., 2019; Vayre and Vonthron, 2019). On the other hand, the possibility to work from home might offer some resources, like time gain due to the elimination of the commute or more flexibility to integrate work and other life domains (Tavares, 2017; Nakrošienė et al., 2019). Past research on job demands and resources in a teleworking setting offers us some indications about which job characteristics are perceived as (dis)advantageous (e.g., Gajendran and Harrison, 2007; Sardeshmukh et al., 2012; Tremblay and Thomsin, 2012; Tavares, 2017). Nevertheless, we neither know which are the most crucial working conditions in such a rapid transition as caused by the pandemic nor how people react to them. Thus, to explore the advantages and disadvantages of working from home during the COVID-19 pandemic, we chose a qualitative approach aimed at the following questions:

**R1:** Which are the top three advantages and disadvantages participants perceive regarding working from home during the pandemic?

Bakker and Demerouti (2017) point out the flexibility of the JD-R model regarding the function/role of job characteristics within the model, highlighting that fewer demands do not automatically equate to resources. In terms of an exceptional situation like the pandemic, this suggests a necessity to re-evaluate the nature and strength of job demands and resources for that particular situation. It is important to note that the open-ended qualitative questions about (dis)advantages allow naming an increase/decrease of both job demands and resources. For example, asked about advantages, participants could name more autonomy on the one hand and less social stress with colleagues on the other hand. Nevertheless, the decrease in a particular straining demand could be seen as especially relieving for people during the pandemic. Thus, we set out to explore how job demands and resources relate to the (dis)advantages perceived by the participants:

**R2:** How do the perceived advantages and disadvantages of working from home relate to specific job characteristics?

Additionally, we want to explore the change people perceive regarding their working conditions before the pandemic compared to during the pandemic. Thus, combining qualitative and quantitative data, we want to explore whether participants who are new to working from home judge their telework job characteristics differently compared to their previous non-telework environment.

**R3:** Do the job characteristics associated with the identified (dis)advantages of working from home differ from the previous work environment for the “telework newcomer” participants due to COVID-19?

To study relationships between job characteristics and well-being within the JD-R framework, past research often draws on work engagement and emotional exhaustion as the main indicators of the motivational and the health impairment-path proposed in the model (e.g., Schaufeli and Bakker, 2004). Work engagement is defined as a positive work-related state of mind that includes vigor, dedication, and absorption and provides people with the energy to face daily job demands (Schaufeli et al., 2006). In contrast, emotional exhaustion, as a core construct of burnout, is a major indicator of psychological health impairment (Maslach and Leiter, 2008). Referring to the motivational path of the JD-R model, we assume that the identified advantages are associated with more work engagement as shown in multiple studies (for a summary see Bakker and Demerouti, 2017; Nielsen et al., 2017). Furthermore, we assume that the perceived advantages reduce psychological costs and should therefore be associated with less emotional exhaustion (Demerouti et al., 2001).

**H1:** The job characteristics derived from the perceived advantages are positively associated with (a) work engagement and negatively associated with (b) emotional exhaustion.

Similarly, we draw on the strain path of the JD-R model proposing that the identified disadvantages are energy-consuming demands that are related to more emotional exhaustion (e.g., Schaufeli and Bakker, 2004). Moreover, since disadvantages can also be reduced or endangered resources (Halbesleben et al., 2014), we assume an association with motivational problems.

**H2:** The job characteristics derived from perceived disadvantages are (a) negatively associated with work engagement and (b) positively associated with emotional exhaustion.

Furthermore, Sardeshmukh et al. (2012) show that teleworking intensity alters job demands and resources and affects well-being outcomes like work engagement and emotional exhaustion. Based on these findings, we assume that more experienced teleworkers handle the situation differently from newcomers, especially when confronted abruptly with the change like in the pandemic. Taking learning and socialization processes into consideration (Maruyama and Tietze, 2012), experienced teleworkers are more likely to have already crafted their work according to their needs and thus, can rely on established work procedures.

**H3:** Experienced teleworkers report (a) significantly higher levels of job characteristics based on the identified advantages and (b) significantly lower levels of job characteristics based on disadvantages than newcomers to the teleworking situation do.

### The Mediating Role of Job Crafting

Job crafting is a behavior employees engage in to change their job to be more in line with their individual needs and interests (Wrzesniewski and Dutton, 2001). In the JD-R model’s framework, job crafting has been implemented as a proactive behavior aiming at increasing job resources and handling job demands (Tims and Bakker, 2010). Employees use job crafting to improve their person-environment fit or proactively reduce misfit (Chen et al., 2014; Lu et al., 2014; Dust and Tims, 2020). In addition, particularly engaged employees use job crafting to further improve their work environment (Lichtenthaler and Fischbach, 2018). Bakker and Demerouti (2017) discuss this as “gain spirals” (p. 276) in the context of job crafting activities. They propose that job resources stimulate motivational processes, which facilitates job crafting activities that lead to more motivation. In line with this reasoning, research indicates that both, particularly advantageous as well as particularly disadvantageous working conditions, stimulate job crafting (Vogt et al., 2015; Dust and Tims, 2020). Therefore, we expect job crafting to mediate both the positive effects of advantages and the negative effect of disadvantages. In an extreme situation like the COVID-19 pandemic, job-crafting activities might become a necessity. People might rely on social and structural job crafting strategies to deal with the pandemic’s extraordinary contextual demand. Moreover, new resources and disappearing demands should motivate employees to craft their jobs. Research in the context of organizational change has shown that job crafting is used in both regular and threatening change contexts (Petrou et al., 2017).

In our study, we focused on resources crafting behaviors and did not include demands crafting behaviors such as reducing hindering demands (Tims et al., 2012). Meta-analytic evidence suggests that especially job crafting strategies aimed at increasing resources (i.e., seeking structural resources and seeking social resources) increase engagement and reduce exhaustion (Rudolph et al., 2017; Lichtenthaler and Fischbach, 2018; Boehnlein and Baum, 2020). In contrast, reducing hindering demands has been identified as a withdrawal or avoidance behavior, which is relatively ineffective in reducing exhaustion and increasing engagement (Tims et al., 2015; Hu et al., 2020). We focused on behavioral crafting rather than cognitive crafting as we aimed to explore actual behavioral changes (Tims et al., 2012). We assumed that thinking about the meaning of one’s job in general would not improve the telecommuting situation, which is the aim of cognitive crafting (Wrzesniewski and Dutton, 2001).

When employees craft their jobs by seeking structural resources, they aim to increase their own competence and autonomy, whereas seeking social resources describes the search for social support (Tims et al., 2012). Resulting job and personal resources such as feelings of self-efficacy or optimism or autonomy and feedback increase engagement (Tims et al., 2013; Vogt et al., 2015). Seeking structural and social resources are both associated with reduced psychological distress (Sakuraya et al., 2017). Bakker and de Vries (2021) suggest that actively seeking structural resources such as new competencies is a form of adaptive self-regulation. It increases personal resources (e.g., self-efficacy, optimism) that facilitate the handling of demands. Social support seeking from supervisors or colleagues is known to be an effective coping strategy to handle exhaustion (Shin et al., 2014). In sum, advantageous job characteristics could be enablers of job crafting activities, while disadvantageous job characteristics provoke the need for job crafting as a coping strategy.

**H4:** Job characteristics based on perceived telework advantages are associated with (a) increased work engagement and (b) lower emotional exhaustion via seeking structural and social job crafting activities.

**H5:** Job characteristics based on perceived telework disadvantages are associated with (a) increased work engagement and (b) lower emotional exhaustion via seeking structural and social job crafting activities.

## Materials and Methods

### Sample

Convenience sampling was used to administer online questionnaires to people who teleworked from home in Germany between March 27 and June 30, 2020, resulting in 750 responses. The questionnaire consisted of two consecutive parts, a qualitative and a quantitative part, and participants could choose to stop after answering the qualitative questions and demographics. For the current study, we draw on the participants who answered both parts of the questionnaire and agreed upon the scientific usage of the data, which resulted in 602 participants. Three participants were deleted due to omissions on both independent variables resulting in a final sample of *N* = 599. Participants were on average 42 years old (*M* = 42.25, *SD* = 10.70), and 34% identified as male, 66% as female, and less than one percent as divers. The majority of 76% reported that they lived with others rather than alone, and 79% indicated to be in a relationship. Care for dependent children was stated by 34% of the participants, 16% of whom indicated having the sole care responsibility. Asked whether they can telework from a separate office only used by them, 31% indicated “yes,” 26% “no,” and 43% reported that the room was used by other people and/or for other purposes. Participants worked on average 37.09 h a week (*SD* = 9.04) and only a minority of 15% reported to be on short-time work. On average, people were about 10 years (*M* = 10.26, *SD* = 9.31) with their current employer. The majority of 62% held a bachelor or master university degree, and 28% reported holding a leadership position. Participants stem from a wide range of branches ranging from medical and technical work to commerce and administration. More than half of the sample (*n* = 321, 54%) stated that they never or only exceptionally had worked at home before the pandemic (newcomers), whereas 278 (46%) stated they had experience.

### Instruments

At the beginning of the questionnaire, there were questions about demographics and occupational context variables. After that, we asked participants about their three most important perceived advantages and disadvantages of working from home. Following, people rated their working conditions and job crafting behaviors. Here, people who indicated that they had not or only worked from home in exceptional circumstances before the COVID-19 crises were classified as “newcomers.” They rated the working conditions and their job crafting behaviors for their current situation at home as well as retrospectively for their regular work setting. People who regularly or always worked from home were classified as “experienced” and only rated their working situation currently at home. A final section in the questionnaire was dedicated to the participants’ well-being.

#### Qualitative Questions

We introduced three open-ended questions, each about the advantages and disadvantages of working from home, which had a limit of 255 characters. The open-ended questions were: “The three biggest advantages I perceive regarding working from home are…” and “the three biggest disadvantages I perceive when working from home are….”

#### Working Conditions

We applied the German Risk Assessment Questionnaire (FGBU, Dettmers and Krause, 2020) to capture work-related risk factors as outlined in the GDA (Gemeinsame Deutsche Arbeitsschutzstrategie, 2017). Here, risk factors are grouped into four categories: work content, work organization, social relations at work, and work environment. Based on the exploratory prioritization via the qualitative answers, we consecutively only report the six quantitative variables from the FGBU used in our manuscript, namely autonomy, interruptions, overtime, lack of information, lack of communication, and work environment. The answering format of all variables ranges from 1 = “strongly disagree” to 4 = “strongly agree.”

*Autonomy* at work was captured with three items, such as “I have a lot of freedom in the way I do my job.” Chronbach’s alpha was 0.80 for the regular work and 0.79 for the home setting.

*Interruptions* were measured with three items, such as “I often have to interrupt a task I am in the process of completing because something urgent comes up.” Chronbach’s alpha was 0.86 for the regular work and 0.83 for the home setting.

*Overtime* was captured with three items, such as “I often need to be available outside of my official working hours.” Chronbach’s alpha was 0.71 for the regular work and 0.75 for the home setting.

*Lack of information* was measured with three items, such as “Documents, information, and data required for my work are often incomplete.” Chronbach’s alpha was 0.79 for the regular work and 0.82 for the home setting.

*Lack of communication* was measured with three items, such as “My workplace lacks opportunities for personal interaction.” Chronbach’s alpha was 0.52 for the regular work and 0.81 for the home setting.

*Work environment* was captured with three items adopted to the telework situation from home due to the COVID-19 pandemic. Participants were asked to answer the following questions: “My workplace provides optimal technical equipment,” “My workplace allows for concentrated and undisturbed working,” “My workplace provides optimal lighting and ergonomic features.” Following Dettmers and Krause (2020), we built an index, since these three different context factors can occur independently.

#### Job Crafting

We used six items applied by Vogt et al. (2015) to capture two different job crafting aspects. Participants answered three questions regarding increasing structural resources (α_*regular*_ = 0.76; α_*home*_ = 0.77; sample item: “I tried to learn new things at work”) and three questions regarding increasing social resources (e.g., ; α_*regular*_ = 0.69; α_*home*_ = 0.71; sample item: “I ask others for feedback on my job performance.”). The answering format ranged from 1 = “I do not agree at all” to 5 = “I totally agree.”

#### Emotional Exhaustion

We measured emotional exhaustion with three focal items (De Cuyper et al., 2012) from the Maslach Burnout Inventory (Maslach et al., 1996). A sample item is “I feel burned out from my work,” and the answering format ranged from 1 = “I do not agree at all” to 5 = “I totally agree.” Chronbach’s alpha was 0.86.

#### Work Engagement

We captured work engagement with three items from the Utrecht Work Engagement Scale (UWES, Schaufeli and Bakker, 2003; Weigelt et al., 2021). Participants were asked to answer two questions capturing vigor (e.g., “I am immersed in my work”) and one item capturing dedication (“I am enthusiastic about my work”) on a five-point scale ranging from 1 = “I do not agree at all” to 5 = “I totally agree.” The scale yielded a Chronbach’s alpha of 0.92.

#### Control Variables

Because of the extraordinary situation caused by the pandemic, we included a set of control variables. Since child care facilities were closed due to COVID-19 during the time of study and women still face the majority of care work (e.g., Väänänen et al., 2004; van Veldhoven and Beijer, 2012; Meyer et al., 2021), we included gender (0 = female, 1 = male) and whether the participants had children or not (0 = no, 1 = yes). Furthermore, we controlled for age (in years) and whether the participants were newcomers to the telework situation or already had experience (0 = newcomer, 1 = experienced). Additionally, we included working hours per week to control the influence of part-time and short-time work (Kleiner et al., 2015).

### Analysis

The first stage of the analysis concerns the explorative approach realized in the qualitative part of our study. Following the recommendations for content analysis (Mayring, 2015; Kuckartz, 2016), the answers on advantages or disadvantages were categorized by a first rater based on an inductive approach. The categorization was discussed within the research group, which resulted in the development of eight categories for advantages and nine categories for disadvantages with a description of inclusion/exclusion criteria for each. After that, the material was rated by two independent raters based on the category system developed. Each unit of meaning was assigned to one category independently of its rank (whether it was the first, second, or third aspect mentioned). Statements that contained more than one unit of meaning were split and assigned. A “not assigned” category includes statements that were off-topic, incomprehensible, or low in frequency (<10). The interrater reliability resulted in an overall Cohen’s Kappa of 0.83 for the advantages and 0.89 for the disadvantages, indicating substantial to perfect agreement. A discussion of the disagreements and a plausibility check resulted in the final categorization displayed in Table 1.

**TABLE 1:** Categories of advantages and disadvantages based on the qualitative analysis and assigned FGBU dimension.

Perceived advantages (*N* = 1681)				

**Category name**	**Count (%)**	**Short description**	**Anchor example**	**FGBU dimension**
Autonomy/flexibility	518 (31%)	Autonomy and flexibility concerning time issues, location, mode of work, organization of breaks and integration of work and home tasks. No external control	“Flexible organization of working time”	Autonomy
No commute	434 (26%)	Saving time, costs, hassles related to commute; more environmentally friendly	“No commute”	(Less) overtime
Life-domain-balance	129 (8%)	More time for family; better work-domain balance; possibility of care work	“Availability for my family”	–
Focused working	338 (20%)	No distractions/interruptions; calm and concentration; better work atmosphere	“More efficient work due to less interruptions”	(Less) interruptions
Productivity and development	88 (5%)	More efficacy, productivity, creativity and inspiration; less (unnecessary) meetings and more time for own work/development	“I am more efficient”	–
Health behavior	69 (4%)	Better nutrition, movement, health protection; psychological easing	“Less infection risk”	–
Work environment	33 (2%)	Better work environment, better ergonomic/technical equipment; accessibility	“Less heat and noise than in the office”	Work environment
Not assigned	72 (4%)	Not relevant for question; not assignable; low frequency (<10)	“I do not have to shave”	–
**Perceived disadvantages (*N* = 1563)**				
Lack of social interactions	405 (26%)	Social, personal, informal interactions with colleagues; social isolation; team cohesion	“I miss my colleagues”	Lack of communication
Professional communication challenges	231 (15%)	Inferior professional communication (more time/effort needed); cooperation challenges; lack of information; digital communication problems	“Higher need to organize (appointments) communication (digital, telephone)”	Information deficit
Work intensification	111 (7%)	Work intensification, less breaks and recovery time; additional work; more time/effort needed due to organizational inefficacy	“More overtime since commute is used for work”	Work intensification
Life-domain-conflict	226 (14%)	No segregation between life domains possible; no psychological work detachment and extended availability	“No segregation between private and work domain”	–
Distractions and interruptions	141 (9%)	Distractions/interruptions; no calm and possibility to concentrate	“Interruptions by the family”	Interruptions
Health behavior	104 (7%)	Inferior nutrition, movement, more psychological strain, trust/appreciation issues with home-office	“Less movement”	–
Self-discipline and motivation	78 (5%)	Motivational problems, less own/organizational structure, less control	“A lot of self-discipline is needed”	–
Work environment	228 (15%)	Inferior work environment, ergonomic/technical equipment; accessibility problems; higher costs	“Technical problems due to disrupted VPN tunnel”	Work environment
Not assigned	39 (2%)	Not relevant for question; not assignable; low frequency (<10)	“I can’t do my work from home”	–
				

At the second stage of the analysis, we drew on the qualitative analysis results to select the working conditions perceived as most central by our participants. Since the categories were built inductively, some did not have an equivalent in the FGBU questionnaire used. Deductively, we matched the specific scientific constructs from the FGBU with the broader qualitative categories.

The third stage concerned quantitative analyses. Applying a paired *t*-test, we compared the selected working conditions and job crafting activities for the “newcomers” subsample. All *t*-tests were subject to Bonferroni corrections. To compare the situation of “newcomers” and “experienced” teleworkers, we applied a MANOVA to test all work characteristics together and interpret the combined results. Investigating the prerequisites of the MANOVA revealed that the dependent variables were not normally distributed and the variable autonomy violated the pre-requisite of homogeneity of error variances (*p* = 0.027) by Levene’s test. All other prerequisites were met. Thus, we continued the MANOVA because it is relatively robust against violations of normal distribution and homogeneity of error variances (e.g., Finch, 2005). To investigate differences between experienced teleworkers and newcomers on specific work characteristics, we conducted *post hoc* univariate ANOVAs.

We then conducted a hierarchical regression analysis to investigate the relationship between the six selected working conditions and work engagement, respectively emotional exhaustion. We entered the control variables in the first step and the job demands and resources as predictors in the second step. Finally, we introduced the two job crafting dimensions as parallel mediators in the analyses applying model 4 of the process macro by Hayes (2018) setting the bootstrapping iterations to 5,000.

## Results

Since our study is composed of a qualitative and a quantitative stage, we first report the results for the research questions and present the subsequent analyses and quantitative results thereafter.

### Results Regarding the Research Questions

Table 1 shows the categories developed with a short description, an anchor example, and their frequency. Overall, participants named 1,681 advantages and 1,563 disadvantages. Addressing **research question one (R1**), the frequencies indicate that enhanced flexibility and autonomy (518, 31%) was perceived as most advantageous, followed by the lack of commute (434, 26%) and more focused working (338, 20%). Participants also mentioned a better integration of life domains (129, 8%), more productivity and development (88, 5%), improved health behavior (69, 4%), and a better work environment (33, 2%). In terms of disadvantages, participants cited lack of social interactions (405, 26%), professional communication challenges (231, 15%), and an inadequate work environment (228, 15%) as the most serious drawbacks. Additionally, they reported life-domain conflicts (226, 14%), distractions, and interruptions (141, 9%), work intensification (11, 7%), impaired health behavior (104, 7%), and challenges regarding self-discipline and motivation (78, 5%).

Relating the identified (dis)advantages to specific working conditions (**research question two, R2**) meets two challenges: first, not all the grouped (dis)advantages concern working conditions. Some rather describe consequences, motivational aspects, or general health behaviors. Second, the categories of the indicated (dis)advantages are more abstract than the scientific constructs measured by the FGBU. Thus, we only selected (dis)advantages that corresponded to one of the dimensions measured by the FGBU and focused on the most salient aspect described in the respective categories. Since we found the categories work environment and interruptions for both advantages and disadvantages, we assigned them as advantage or disadvantage upon their frequencies. Thus, we were able to allocate the FGBU-dimensions autonomy, (lack of) interruptions, and (lack of) overtime to the advantages associated with working from home (see Table 1). It is important to note that only autonomy is a resource (Dettmers and Krause, 2020), whereas the other two advantages describe a reduction of demands. In terms of job disadvantages, lack of communication, information deficit, and an inadequate work environment were the three major challenges. We operationalized the (dis)advantages according to the polarity of the FGBU for further analysis.

Based on the operationalization of the (dis)advantages with the constructs from the FGBU, we found significant differences regarding all six job characteristics (**research question three, R3**) for newcomers to teleworking due to COVID-19. The three identified advantages of working from home (autonomy, less overtime, fewer interruptions, see Table 2) were evaluated more favorably in the telework situation than in the usual work setting. Likewise, the identified disadvantages correspond with less favorable ratings regarding the dimensions lack of communication, information deficit, and inadequate work environment (see Table 2). Interestingly, newcomers also indicated significantly fewer job crafting activities at home than in their usual work setting.

**TABLE 2:** Comparison between the job conditions and job crafting behaviors for newcomers and experienced teleworkers.

	Telework newcomers					Telework newcomers versus experienced teleworkers				
Job condition	Workplace *M (SD)*	Telework *M (SD)*	Paired *t*-test *t* (df)	*p*	Cohen’s d | *d*|	Newcomers *M (SD)*	Experienced *M (SD)*	ANOVA*F*(1,591)	*p*	Effect size ω^2^
Autonomy	3.04 (0.73)	3.14 (0.73)	4.86 (409)	<0.001	0.14	3.10 (0.76)	3.30 (0.65)	10.84	=0.001	0.02
Overtime^a^	1.99 (0.77)	1.90 (0.83)	3.00 (409)	=0.021	0.11	1.90 (0.84)	2.03 (0.84)	3.34	=0.068	<0.01
Interruptions^a^	2.97 (0.84)	2.20 (0.79)	18.47 (409)	<0.001	0.94	2.18 (0.80)	2.53 (0.87)	23.76	<0.001	0.04
Lack of communication	1.17 (0.38)	1.85 (0.86)	−16.22 (403)	<0.001	1.02	1.88 (0.86)	1.87 (0.85)	0.04	=0.850	<0.01
Work environment^b^	3.08 (0.67)	2.68 (0.75)	10.06 (409)	<0.001	0.56	2.65 (0.76)	2.84 (0.80)	8.53	=0.004	0.01
Information deficit	2.35 (0.76)	2.71 (0.81)	−13.31 (409)	<0.001	0.46	2.74 (0.83)	2.52 (0.84)	11.54	=0.001	0.02
Job crafting CSR	4.12 (0.84)	4.04 (0.90)	3.14 (409)	<0.001	0.09	4.02 (0.93)	4.25 (0.82)	9.87	=0.002	0.02
Job crafting CSOR	3.07 (0.97)	2.74 (0.97)	12.50 (409)	=0.014	0.34	2.71 (0.97)	3.03 (0.99)	12.97	<0.001	0.02

*p = Bonferroni corrected for paired t-tests.*

*^a^Worded as advantage but coded reversely in the FGBU.*

*^b^Worded as disadvantage but coded reversely in the FGBU.*

### Results Regarding the Hypotheses

Means, standard divisions, reliabilities, and correlations of all study variables are displayed in Table 3.

**TABLE 3:** Means, standard deviations, correlations, and internal consistencies for all study variables.

		*M*	*SD*	1	2	3	4	5	6	7	8	9	10	11	12	13	14	15
1	Gender	−	−	−	0.00	0.00	0.00	0.00	0.00	0.00	0.00	0.00	0.00	0.00	0.00	0.00	0.00	0.00
2	Age	42.25	10.70	0.19**	−	0.00	0.00	0.00	0.00	0.00	0.00	0.00	0.00	0.00	0.00	0.00	0.00	0.00
3	Children	−	−	0.02	–0.01	−	0.00	0.00	0.00	0.00	0.00	0.00	0.00	0.00	0.00	0.00	0.00	0.00
4	Working time (h)	37.09	9.04	0.29**	0.07	−0.15**	−	0.00	0.00	0.00	0.00	0.00	0.00	0.00	0.00	0.00	0.00	0.00
5	Newcomer	−	−	0.00	0.01	0.08	–0.00	−	0.00	0.00	0.00	0.00	0.00	0.00	0.00	0.00	0.00	0.00
6	Autonomy	3.19	0.72	0.11*	0.05	0.02	0.13**	0.14**	0.79	0.00	0.00	0.00	0.00	0.00	0.00	0.00	0.00	0.00
7	Overtime^a^	1.96	0.84	0.07	–0.03	0.06	0.31**	0.08	0.08	0.75	0.00	0.00	0.00	0.00	0.00	0.00	0.00	0.00
8	Interruptions^a^	2.56	1.38	0.07	–0.04	0.22**	0.21**	0.20**	0.20**	0.46**	0.83	0.00	0.00	0.00	0.00	0.00	0.00	0.00
9	Lack of communication	1.86	0.87	0.03	0.12**	–0.02	–0.01	0.01	0.15**	0.03	−0.13**	0.81	0.00	0.00	0.00	0.00	0.00	0.00
10	Work environment^b^	2.73	0.79	0.09*	0.09*	−0.14**	0.13**	0.12**	0.25**	–0.03	–0.04	–0.04	−	0.00	0.00	0.00	0.00	0.00
11	Information deficit	2.64	0.84	0.05	0.02	0.02	0.12**	−0.13**	0.11**	0.23**	0.24**	0.07	–0.03	0.82	0.00	0.00	0.00	0.00
12	Job crafting CSR	4.13	0.89	0.06	0.06	0.05	0.11**	0.13**	0.46**	0.20**	0.30**	–0.06	0.38**	0.14**	0.77	0.00	0.00	0.00
13	Job crafting CSOR	2.85	0.99	0.02	−0.10*	0.07	0.10*	0.15**	0.13**	0.19**	0.28**	−0.16**	0.18**	0.08*	0.43**	0.71	0.00	0.00
14	Emotional exhaustion	2.12	0.97	–0.06	−0.14**	0.06	–0.02	–0.04	−0.17**	0.21**	0.12**	–0.01	−0.26**	0.19**	−0.24**	−0.14**	0.86	0.00
15	Work engagement	3.31	0.96	0.08	–0.10	0.08*	0.07	0.06	0.16**	0.13**	0.07	–0.08	0.21**	–0.07	0.39**	0.28**	−0.36**	0.92

*N = 593–599.*

*^a^Worded as advantage but coded reversely in the FGBU.*

*^b^Worded as disadvantage but coded reversely in the FGBU.*

*p < 0.05; **p < 0.01.

*All job characteristics refer to the telework situation.*

*Chronbach’s alpha is displayed in the diagonal.*

*Gender: 0 = female, 1 = male; children: 0 = no, 1 = yes; newcomer: 0 = newcomer, 1 = experienced.*

*CSR, increasing structural resources; CSOR, increasing social resources.*

Overall, the results of the regression analyses show that all the predictors together explain 11.4% [*R*^2^ = 0.11; *F*(11,566) = 6.61, *p* < 0.001] of the variance in work engagement and 15.4% [*R*^2^ = 0.15; *F*(11,567) = 9.39, *p* < 0.001] of the variance in emotional exhaustion (see Table 4).

**TABLE 4:** Hierarchical regression analyses on work engagement and emotional exhaustion.

		Work engagement							Emotional exhaustion						
		*B*	95% CI for *B*		SE *B*	β	*Adj. R* ^2^	Δ*R*^2^	*B*	95% CI for *B*		SE *B*	β	*Adj. R* ^2^	Δ*R*^2^
Step and predictor			LL	UL						LL	UL				
1	Constant	2.57	2.09	3.05	0.24	0.00	0.02	0.03**	2.50	2.02	2.992	0.25	0.00	0.02*	0.03*
	Gender	0.09	–0.08	0.26	0.09	0.05	0.00	0.00	–0.09	–0.26	0.09	0.09	–0.04	0.00	0.00
	Age	0.01	0.00	0.02	0.00	0.09*	0.00	0.00	–0.01	–0.02	–0.00	0.00	−0.12**	0.00	0.00
	Children	0.18	0.01	0.34	0.09	0.09*	0.00	0.00	0.16	–0.02	0.33	0.09	0.08	0.00	0.00
	Working time	0.01	–0.00	0.02	0.01	0.07	0.00	0.00	0.00	–0.01	0.01	0.01	0.03	0.00	0.00
	Newcomer	0.10	–0.06	0.26	0.08	0.05	0.00	0.00	–0.10	–0.26	0.06	0.08	–0.05	0.00	0.00
2	Constant	1.59	0.89	2.29	0.36	0.00	0.10	0.09**	2.97	2.29	3.66	0.35	0.00	0.14**	0.13**
	Gender	0.07	–0.10	0.23	0.08	0.03	0.00	0.00	–0.05	–0.21	0.12	0.08	–0.02	0.00	0.00
	Age	0.01	0.00	0.02	0.00	0.09*	0.00	0.00	–0.01	–0.02	–0.00	0.00	−0.10*	0.00	0.00
	Children	0.20	0.03	0.36	0.09	0.10*	0.00	0.00	0.03	–0.13	0.20	0.08	0.02	0.00	0.00
	Working time	0.00	–0.01	0.01	0.01	0.00	0.00	0.00	–0.00	–0.01	0.01	0.01	–0.03	0.00	0.00
	Newcomer	–0.03	–0.19	0.13	0.08	–0.02	0.00	0.00	0.00	–0.16	0.16	0.08	0.00	0.00	0.00
	Autonomy	0.21	0.09	0.33	0.06	0.14**	0.00	0.00	–0.19	–0.31	–0.07	0.06	−0.12**	0.00	0.00
	Overtime^a^	0.18	0.08	0.28	0.05	0.16**	0.00	0.00	0.20	0.10	0.30	0.05	0.17**	0.00	0.00
	Interruptions^a^	–0.01	–0.12	0.10	0.06	–0.01	0.00	0.00	0.02	–0.08	0.13	0.06	0.02	0.00	0.00
	Lack of communication	–0.10	–0.19	–0.01	0.05	−0.09*	0.00	0.00	0.00	–0.09	0.09	0.05	0.00	0.00	0.00
	Work environment^b^	0.25	0.15	0.36	0.05	0.20**	0.00	0.00	–0.24	–0.35	–0.14	0.05	−0.19**	0.00	0.00
	Information deficit	–0.09	–0.19	0.00	0.05	–0.08	0.00	0.00	0.18	0.08	0.28	0.05	0.15**	0.00	0.00

*N = 593–599.*

*Worded as advantage but coded reversely in the FGBU.*

*^b^Worded as disadvantage but coded reversely in the FGBU.*

*p < 0.05; **p < 0.01.

*Gender: 0 = female, 1 = male; children: 0 = no, 1 = yes; newcomer: 0 = newcomer, 1 = experienced.*

*CI, confidence interval; LL, lower limit; UL, upper limit.*

Concerning hypothesis 1a regarding the positive association of the identified advantages with work engagement, only autonomy (β = 0.14, *p* = 0.001) is a significant positive predictor. The participants cited less overtime as an advantage, but overtime -operationalized as a risk factor in the FGBU- was actually positively associated with work engagement (β = 0.16, *p* = 0.001). Fewer interruptions did not prove to be associated with either work engagement or emotional exhaustion (see Table 4).

Concerning hypothesis 1b regarding the negative association of the identified advantages with emotional exhaustion, we found the predictors autonomy (β = −0.12, *p* = 0.002) and overtime (β = 0.17, *p* < 0.001) to be in line with our hypothesis.

Concerning hypothesis 2a regarding the negative association of the identified disadvantages with work engagement, we found support regarding lack of communication (β = −0.09, *p* = 0.033) and work environment (reverse coded; β = 0.20, *p* < 0.001). No significant association could be shown for information deficit (see Table 4).

Concerning hypothesis 2b regarding the positive association of the identified disadvantages with emotional exhaustion, we found support regarding information deficit (β = 0.15, *p* < 0.001) and work environment (reverse coded; β = −0.19, *p* < 0.001). No significant association could be shown for lack of communication (see Table 4).

The one-way MANOVA addressing hypotheses 3a and b revealed significant differences between experienced teleworkers and telework newcomers on the combined job characteristics [*F*(8,584) = 8.528, *p* < 0.001, partial ω^2^ = 0.092, Wilk’s Λ = 0.895].

Concerning hypothesis 3a, the *post hoc* univariate ANOVAs showed that experienced teleworkers indeed reported more autonomy than people new to the situation [*F*(1,591) = 10.84, *p* = 0.001, ω^2^ = 0.02], but no significant differences could be shown for overtime (see Table 2). Contrary to our assumptions, experienced teleworkers indicated significantly more interruptions than newcomers [*F*(1,591) = 23.76, *p* < 0.001, ω^2^ = 0.04].

Concerning hypothesis 3b, the *post hoc* univariate ANOVAs show that experienced teleworkers indeed reported significantly fewer problems with an inadequate work environment [*F*(1,591) = 8.53, *p* = 0.004, ω^2^ = 0.01] and information deficits [*F*(1,591) = 11.54, *p* = 0.001, ω^2^ = 0.02]. In contrast, no difference could be shown for lack of communication (see Table 2).

The results regarding the mediations in hypotheses 4 and 5 are displayed in Table 5. In terms of the advantages, the job crafting behaviors mediated all relationships between the three job characteristics (autonomy, overtime, interruption) and emotional exhaustion, as well as work engagement, except for seeking social resources, which was no mediator between autonomy and the two outcomes. A closer look at the a-path of the mediation models revealed that increased autonomy was positively associated with seeking structural resources, whereas less overtime and fewer interruptions were negatively related to both job-crafting activities.

**TABLE 5:** Direct and indirect effects of job characteristics on work engagement (WE) and emotional exhaustion (MBI) via job crafting structural (CSR) and social resources (CSOR).

Independent	Outcome	Direct effect	CSR	CSOR
Autonomy	WE	0.10 [−0.01; 0.22]	**0.12** [0.07; 0.19]	−0.01 [−0.03; 0.01]
	MBI	−**0.16** [−0.29; −0.04]	−**0.06** [−0.12; −0.02]	0.00 [−0.01; 0.02]
Overtime^a^	WE	0.06 [−0.03; 0.15]	**0.04** [0.01; 0.08]	**0.02** [0.00; 0.05]
	MBI	**0.32** [0.22; 0.41]	−**0.03** [−0.06; −0.01]	−**0.02** [−0.04; −0.00]
Interruptions^a^	WE	−0.08 [−0.17; 0.01]	**0.07** [0.04; 0.11]	**0.04** [0.02; 0.07]
	MBI	**0.24** [0.14; 0.34]	−**0.05** [−0.08; −0.02]	−**0.02** [−0.05; −0.00]
Lack of communication	WE	−0.00 [−0.09; 0.08]	−**0.06** [−0.10; −0.02]	−**0.04** [−0.06; −0.02]
	MBI	−0.04 [−0.13; 0.05]	**0.03** [0.01; 0.06]	**0.02** [0.00; 0.04]
Work environment^b^	WE	**0.16** [0.06; 0.26]	**0.09** [0.05; 0.13]	**0.02** [0.00; 0.04]
	MBI	**0.24** [0.14; 0.34]	−**0.05** [−0.08; −0.02]	−0.01 [−0.03; 0.00]
Information deficit	WE	−**0.09** [−0.18; −0.00]	−0.01 [−0.05; 0.02]	0.00 [−0.02; 0.02]
	MBI	**0.25** [0.15; 0.34]	0.00 [−0.02; 0.02]	0.00 [−0.01; 0.01]

*N = 580–581.*

*^a^Worded as advantage but coded reversely in the FGBU.*

*^b^Worded as disadvantage but coded reversely in the FGBU.*

*Shown are estimates of the direct and indirect effects, and 95% confidence intervals in brackets, bootstrap iterations = 5,000; significant indirect effects in bold; controlled for gender, age, children, working hours, and newcomer status.*

Regarding disadvantages, seeking structural and social resources mediated between lack of communication and emotional exhaustion, as well as work engagement (see Table 5). The same results were achieved for the independent variable work environment except for seeking social resources, which did not mediate between work environment and work engagement. No mediation could be shown for information deficit. For lack of communication and an inadequate work environment, the a-path in the mediation revealed a negative relationship with job crafting activities. Thus, the results partially support hypotheses 4 and 5.

## Discussion

The aim of our study was to investigate how people perceive and react to an abrupt change in working conditions as caused by the COVID-19 pandemic. Our results show that people who had to change from their usual work setting to working from home report significant differences in their working conditions. To address our first research question (R1) on the three most frequently voiced advantages and disadvantages in working from home, we applied a qualitative approach. In this way, we identified autonomy, fewer interruptions, and less overtime as the most frequently described advantages and communication problems, information deficits, and inadequate work environment as the three most central disadvantages. Based on these qualitative results, we were able to match the corresponding quantitative data from the FGBU questionnaire (Dettmers and Krause, 2020; R2). The quantitative measures indicated significant changes in all six job characteristics for telework newcomers as compared to the pre-pandemic work setting (R3). Congruent with the qualitative data, the identified advantages were evaluated more and the disadvantages less favorably in the telework setting.

Concerning the identified advantages of working from home, our quantitative results confirm autonomy to be a central resource. As assumed in our first hypothesis autonomy is significantly associated with more work engagement and less emotional exhaustion. This result highlights the outstanding importance of autonomy as a resource, as shown by previous research (Halbesleben et al., 2014; Ryan and Deci, 2017; Meyer et al., 2021).

Furthermore, our participants reported less overtime as a clear advantage of working from home, and our quantitative results indicate that less overtime is indeed associated with less emotional exhaustion. For the motivational aspect of work engagement, we found – contrary to our hypothesis 1, that less overtime is associated with less work engagement. Overtime, just as time pressure, may act as a challenge demand that motivates employees by being challenging (LePine et al., 2005). Employees who experience time pressure at the day-level are more engaged, but only if their job control is high (Kühnel et al., 2012). Moreover, job crafting research indicates that employees actively increase challenge demands crafting to improve their motivation (Petrou et al., 2012). Nevertheless, it seems important to stress that our study is cross-sectional, and reverse or reciprocal effects might apply, such as less engaged people might be less motivated to overtime.

For the third advantage mentioned, “fewer interruptions,” we found no significant associations with work engagement or emotional exhaustion. It is worth mentioning that overtime and interruptions are operationalized as psychological risk factors in the FGBU and not as resources (Dettmers and Krause, 2020). Thus, even though our participants consider them advantageous, they might not qualify as job resources in telework, but rather as reduced demands (Bakker and Demerouti, 2017).

Addressing hypothesis 2 on the reported disadvantages, our quantitative data shows that an inadequate work environment was significantly associated with emotional exhaustion and less work engagement. Lack of communication was related only to less work engagement but not to emotional exhaustion, whereas information deficit was related only to more emotional exhaustion. These findings are in line with previous research on social relatedness, in this study measured reversely as “lack of communication,” as a central psychological resource (Ryan and Deci, 2017) stimulating the motivational path outlined in the JD-R (Bakker and Demerouti, 2017; Ryan and Deci, 2017). By contrast, “information deficit” is a classic job demand that drains energy and causes psychological strain, as outlined in the JD-R strain path (Dettmers and Krause, 2020).

To get a clearer picture of how people adapt to telework situations in the pandemic, we compared more experienced teleworkers with newcomers (hypothesis 3). Consistent with our assumptions, we found that experienced teleworkers report more autonomy, fewer information deficits, and a more appropriate work environment. These results suggest that people seem to shape teleworking conditions accordingly over time, just as they change job characteristics in general by using job crafting behavior (Tims et al., 2013). Our results underscored this notion showing that experienced teleworkers reported significantly more job crafting activities. However, experienced teleworkers did not report less overtime or improved communication, and they reported more interruptions than the newcomers. One explanation could be that experienced teleworkers continued their “normal” work routines during the pandemic, characterized by typical daily interruptions. Newcomers, by contrast, might have been busy reorganizing their own work during this initial phase of the pandemic including, e.g., managing the technical setup. Since their work routines had not been established, they might have experienced fewer interruptions by co-workers or supervisors.

Then again, newcomers initially reported fewer job crafting activities in the telework situation than in the previous work setting. Thus, it seems possible that (a) some working conditions are not easy to address by the teleworking employees themselves but might have to be organized at an organizational level, (b) that job crafting as a proactive behavior is subject to an adaptation process and newcomers were still in an initial orientation phase.

Regarding the mediating role of the job crafting activities, we proposed in hypothesis 4 for the identified advantages, we found that increasing structural resources to be an important mechanism to increase work engagement and decrease emotional exhaustion. With respect to overtime and interruptions, participants also sought social job resources to foster work engagement and reduce emotional exhaustion. For autonomy, we found no mediating role of increasing social job resources. Here, it seems important to note that autonomy is the only “classical” resource named among the three advantages in our study, whereas participants appreciate less overtime and fewer interruptions in the telework setting. Thus, the latter two are reduced demands rather than actual resources. Nevertheless, both demands, overtime and interruptions, are positively related to seeking structural and social resources. These results are consistent with findings that indicate that advantageous as well as disadvantageous work characteristics might stimulate job crafting activities (Vogt et al., 2015; Dust and Tims, 2020). In our study, it seems that the surplus of autonomy in the telework situation was used to seek structural resources, whereas the relief from overtime and interruptions might have made other job crafting activities less urgent. Interestingly, seeking structural resources fully mediated the relationship between autonomy, overtime, and interruptions with work engagement. This is an unusual finding, as the direct effect between job resources, job demands and well-being is well documented (Bakker and Demerouti, 2017). However, these findings apply to the office context. Telework and the pandemic context might again play a particular role making the need to proactively shape one’s work setting to stay motivated more salient.

Hypothesis 5 concerned the mediating role of job crafting activities between the disadvantages and emotional exhaustion as well as work engagement. We found job crafting activities to be relevant mediators for work environment as well as lack of communication. An inadequate work environment and lack of communication were negatively related to the two job-crafting dimensions and both were named as particularly burdensome when working from home. Furthermore, information deficit was found to be entirely unrelated to job crafting activities. Thus, compared to overtime and interruption, which we addressed as potential triggers for job crafting activities, an inadequate work environment, lacking communication, and information deficit seem to hinder them. Again, this might be due to the specifics of the teleworking demands during the pandemic: more autonomy might provide leeway to proactively address certain demands such as overtime or interruptions (particularly, if not urgent). For example, Breevaart and Tims (2019) showed that employees were particularly engaged in seeking structural resources on days when they experienced high levels of resources and when their perceived job insecurity (e.g., fear of losing their job) was low. This might also explain the higher levels of job crafting among experienced teleworkers, who are likely to feel less insecure. Then again, resources like autonomy might not have the strength to substitute more pressing demands such as an inadequate work environment or lack of communication. Here, much more research is needed to differentiate between job demands in the telework setting and investigate the interplay between job resources and demands in terms of job crafting activities.

### Implications for Research and Practice

The sudden transition to working from home caused by the pandemic reveals the necessity to investigate the telework socialization processes. Our findings show that working conditions are evaluated differently in telework compared to the previous work setting and that adjustment processes are likely to occur. Longitudinal studies are needed to examine the drivers of these adjustment processes as well as potential moderating and mediating factors. For instance, based on the propositions made by JD-R (Bakker and Demerouti, 2017), it would be interesting to investigate whether the postulated job resources highlighted in the teleworking setting potentially “buffer” the strain associated with job demands.

Furthermore, it would be important to study a wider range of job crafting activities and coping behaviors. Here, it would be interesting to investigate how job characteristics relate to job crafting activities. For instance, are resources a prerequisite for engaging in job crafting, or are these behaviors a response to job demands – or both? Employees use job crafting to improve person-job fit (Chen et al., 2014; Dust and Tims, 2020). Nevertheless, research on the role of job crafting for telework is scarce. As some job crafting behaviors are known to be less advantageous but associated with high exhaustion (Lichtenthaler and Fischbach, 2018), it is necessary to explore further job crafting behaviors. In this study, we focus on crafting behaviors addressing job resources. Job crafting activities aiming at job demands might be easier for newcomers in the ambiguity of a new work situation.

Our study contributes to research on job resources and job demands because it does not take common categorizations of job demands and resources as given but rather applies a multi-method approach to assess the importance of the specific teleworking condition. This is essential because the most relevant job characteristics for employee well-being in the telework context are still not sufficiently understood. Thus, our results feed into the ongoing theoretical debate on whether the categorization of job characteristics as demands or resources depends on individual appraisal (Bakker and Demerouti, 2017). The qualitative results of our study show that people indeed perceive a lack of demands as beneficial or a lack of a resource as detrimental. Here, it seems worthwhile to investigate thresholds related to these job characteristics to more clearly conceptualize whether a lack of a demand can constitute a resource and vice versa (Bakker and Demerouti, 2017).

Furthermore, the qualitative results suggest that there are differential preferences regarding working conditions, such as integration versus segregation of life domains. Based on theoretical frameworks like boundary theory (Ashforth et al., 2000) or the work-home resources model (ten Brummelhuis and Bakker, 2012) a deeper investigation of the different needs and preferences at the interface between life domains and their influence on well-being could help to further research on (tele)work.

Several practical implications can be derived from our study. First, the qualitative analyses of the (dis)advantages of working from home reveal the working conditions that are perceived as most central to the current telework situation. Thus, increasing autonomy, fostering communication, and providing an adequate work environment and information while actively reducing interruptions and overtime seem to be appropriate starting points for designing telework.

Second, differences based on telework experience suggest that organizations need to facilitate and promote transition processes. This could be done by, for example, establishing an onboarding workshop for teleworking to inform people about potential challenges, risk factors, and expectations. Furthermore, organizations should establish strategies to ensure an adequate and safe work environment.

Third, encouraging job crafting behaviors, especially among newcomers, could help improve very individualized telework spaces. Both leadership behavior (Hetland et al., 2018) and job crafting interventions (Oprea et al., 2019) can successfully increase job crafting behavior. Furthermore, how organizations communicate in situations of crises or change might be important. Research shows that adequate change communication increases work engagement, for example, by seeking structural resources (Petrou et al., 2018). Future research should include additional job crafting behaviors, such as avoidance crafting or other crafting approaches, such as cognitive crafting, as potential facilitators of change and transition. Recent research shows that avoidance crafting might be useful when combined with approach crafting (Petrou and Xanthopoulou, 2020). In telework contexts with less leadership guidance, an effective combination of avoiding irrelevant demands and approaching relevant ones might become more important. Cognitive crafting might be particularly useful in contexts where employees have only little autonomy when and how to do telework, for example when homeschooling needs to be managed in parallel. Here, it can increase experienced meaningfulness, an important predictor of motivation (Geldenhuys et al., 2021).

### Strength and Limitations

Our study focuses on working conditions during the COVID-19 pandemic providing valuable insights not only into working conditions in telework but also into abrupt transitions to telework. Nevertheless, the reported study results are only cross-sectional. For example, we cannot rule out the possibility that job demands and resources mediate the relationship between job crafting and well-being and not vice versa. However, as job resources, job crafting, and work engagement affect each other positively as part of a gain spiral, we assume that this alternative does not reduce the relevance of the results. The co-occurrence of these factors is representative of a motivating work environment (Bakker and Demerouti, 2017). Therefore, we can draw no conclusions about causality, and the mediation effects should be validated longitudinally.

The specificity of the COVID-19 situation raises questions about the generalizability of our results beyond the pandemic. Generally, we assume that our findings about the facilitating role of job crafting are applicable during other change or transition processes as they are in line with other findings about the role of job crafting in change processes (e.g., Petrou et al., 2017, 2018). However, we assume that the pandemic caused radical changes in the telework culture in many countries and that future research will need to show the extent to which telework research before and during the pandemic is applicable to the future. In particular, we believe that the vast majority of employees working in jobs that theoretically allow telework had at least some telework experience during the pandemic. Apart from newcomers on the job marked, we are unlikely to have many opportunities to study telework newbies in the near future. We therefore especially support requests to investigate telework conditions beyond the pandemic (see, e.g., Contreras et al., 2020).

The working conditions before the pandemic were evaluated retrospectively and only for telework newcomers and thus may be subject to perceptional or memory biases. A low reliability concerning the measure *lack of communication* for the pre-pandemic evaluation additionally limits interpretability. Here, longitudinal research is needed to investigate changes and transition processes.

By providing a combination of qualitative and quantitative data, we are able to draw a more comprehensive picture of the (dis)advantages perceived regarding telework. For example, the mixed-method approach revealed that a reduction of demands is indeed seen as advantageous in our sample. However, participant responses encompass a much greater variety than we captured with our quantitative measure. Therefore, selecting and focusing on a small number of working conditions and classifying them can only be an approximation and limits the replicability of our study. For instance, the responses regarding “lack of social interactions” were quite broad and by matching them to the quantitative scale “lack of communication,” we focused on the emphasis made in the open statements. From past research, we know that social isolation due to different shortcomings in the social interaction is a common deficiency of telework (Charalampous et al., 2019). Thus, to obtain a more differentiated picture, it would be important to consider further social aspects like for instance “social support by the colleagues.” Nevertheless, except for the categories “no commute” and “life-domain balance,” which may be especially relevant job characteristics in the telework context, the identified advantages, and disadvantages align very well with job demands and resources that have been identified as crucial for employee well-being in the past (compare e.g., Nahrgang et al., 2011; van den Broeck et al., 2017). This supports that the FGBU was suitable to assess the most relevant job characteristics. However, the role of advantages and disadvantages not captured in the FGBU, such as reduced commuting time, should be considered for further telework research.

Convenience sampling via an online questionnaire resulted in a heterogeneous sample that reflects a wide range of occupational backgrounds. However, the emphasis on telework naturally excludes people who are either not allowed to work from home by their employer or simply do not have the possibility to perform their profession from home.

## Conclusion

The COVID-19 pandemic continues to draw much attention to changing work conditions and telework. Our findings shed light on the perceived (dis)advantages of telework and show that people use job crafting activities to shape their work, which in turn influences their well-being. The societal development suggests that telework might be an integral part of our future working lives (Backhaus et al., 2020). Thus, research needs to address and substantiate this development in order to design a healthy and sustainable work environment.

## Data Availability Statement

The raw data supporting the conclusions of this article will be made available by the authors, without undue reservation.

## Ethics Statement

Ethical review and approval was not required for the study on human participants in accordance with the local legislation and institutional requirements. The patients/participants provided their written informed consent to participate in this study.

## Author Contributions

CS initiated the research and made the first draft of the manuscript and also coordinated and performed the qualitative and quantitative analysis. KS contributed by substantiating the theoretical section, and the discussion with literature on job crafting. Both authors have done several revisions of the original draft. Both authors contributed to the article and approved the submitted version.

## Conflict of Interest

The authors declare that the research was conducted in the absence of any commercial or financial relationships that could be construed as a potential conflict of interest.

## Publisher’s Note

All claims expressed in this article are solely those of the authors and do not necessarily represent those of their affiliated organizations, or those of the publisher, the editors and the reviewers. Any product that may be evaluated in this article, or claim that may be made by its manufacturer, is not guaranteed or endorsed by the publisher.
