# Supplementary material for: Suddenly Telework: Job Crafting as a Way to Promote Employee Well-Being?
Source: Front Psychol. 2022 Jan 14;12:790862. doi: 10.3389/fpsyg.2021.790862 (PMC8795870; doi:10.3389/fpsyg.2021.790862)
Supplement: Supplementary file 1 [file Data_Sheet_1.docx]

**Supplemental material**

**SuppM1.**

Means, standard deviations, correlations, and internal consistencies for all study variables in the overall sample based on listwise deletion

|  |  | M | SD | 1 | 2 | 3 | 4 | 5 | 6 | 7 | 8 | 9 | 10 | 11 | 12 | 13 | 14 | 15 |
| --- | --- | --- | --- | --- | --- | --- | --- | --- | --- | --- | --- | --- | --- | --- | --- | --- | --- | --- |
| 1 | Gender^a^ | 0.34 | 0.47 | - |  |  |  |  |  |  |  |  |  |  |  |  |  |  |
| 2 | Age | 42.25 | 10.70 | .18^**^ | - |  |  |  |  |  |  |  |  |  |  |  |  |  |
| 3 | Children^b^ | 0.34 | 0.47 | .02 | -.00 | - |  |  |  |  |  |  |  |  |  |  |  |  |
| 4 | Working time (hrs) | 37.09 | 9.04 | .28^**^ | .05 | -.17^**^ | - |  |  |  |  |  |  |  |  |  |  |  |
| 5 | Newcomer^c^ | 0.46 | 0.50 | -.01 | .01 | .08^*^ | -.02 | - |  |  |  |  |  |  |  |  |  |  |
| 6 | Autonomy | 3.19 | 0.72 | .07 | .03 | .03 | .07 | .14^**^ | (.79) |  |  |  |  |  |  |  |  |  |
| 7 | Overwork ^adv^ | 1.96 | 0.84 | .06 | -.04 | .07 | .29^**^ | .07 | -.01 | (.75) |  |  |  |  |  |  |  |  |
| 8 | Interruptions ^adv^ | 2.56 | 1.38 | .05 | -.05 | .23^**^ | .17^**^ | .20^**^ | .10^*^ | .44^**^ | (.83) |  |  |  |  |  |  |  |
| 9 | Lack of Communication | 1.86 | 0.87 | .03 | .11^**^ | -.01 | -.03 | .00 | .11^**^ | .00 | -.16^**^ | (.81) |  |  |  |  |  |  |
| 10 | Work environment ^dis^ | 2.73 | 0.79 | .07 | .08 | -.14^**^ | .09^*^ | .12^**^ | .10^*^ | -.11^*^ | -.14^**^ | -.08^*^ |  |  |  |  |  |  |
| 11 | Information deficit | 2.64 | 0.84 | .03 | .01 | .03 | .09^*^ | -.15^**^ | -.04 | .18^**^ | .19^**^ | .03 | -.15^**^ | (.82) |  |  |  |  |
| 12 | Job crafting csr | 4.13 | 0.89 | .02 | .05 | .07 | .03 | .14^**^ | .27^**^ | .13^**^ | .21^**^ | -.15^**^ | .23^**^ | -.04 | (.77) |  |  |  |
| 13 | Job crafting csor | 2.85 | 0.99 | .00 | -.11^**^ | .08 | .06 | .15^**^ | -.00 | .15^**^ | .22^**^ | -.20^**^ | .09^*^ | -.01 | .33^**^ | (.71) |  |  |
| 14 | Emotional exhaustion | 2.12 | 0.97 | -.06 | -.13^**^ | .07 | -.00 | -.04 | -.15^**^ | .22^**^ | .14^**^ | -.00 | -.25^**^ | .22^**^ | -.22^**^ | -.13^**^ | (.86) |  |
| 15 | Work engagement | 3.31 | 0.96 | .08 | .10^*^ | .08 | .07 | .06 | .16^**^ | .12^**^ | .07 | -.08 | .21^**^ | -.08 | .41^**^ | .29^**^ | -.36^**^ | (.92) |

*Note. N* = 578 based on listwise deletion; all job characteristics refer to the telework situation; ^adv^ = worded as advantage but coded reversely in the FGBU; ^dis^ = worded as disadvantage but coded reversely in the FGBU; csr = increasing structural resources; csor = increasing social resources; ^a^ 0 = female, 1 = male; ^b^ 0 = no, 1 = yes; ^c^ 0 = newcomer, 1 = experienced; Chronbach’s alpha is displayed in the diagonal; ^*^*p* < .05; ^**^*p* < .01.

**SuppM 2**

*Means, standard deviations, correlations and internal consistencies for all study variables in the subsample changing to telework during the pandemic*

|  |  | M | SD | 1 | 2 | 3 | 4 | 5 | 6 | 7 | 8 | 9 | 10 | 11 | 12 | 13 | 14 | 15 | 16 | 17 | 18 | 19 | 20 | 21 | 22 |
| --- | --- | --- | --- | --- | --- | --- | --- | --- | --- | --- | --- | --- | --- | --- | --- | --- | --- | --- | --- | --- | --- | --- | --- | --- | --- |
| 1 | Gender^a^ | 0.35 | 0.48 | - |  |  |  |  |  |  |  |  |  |  |  |  |  |  |  |  |  |  |  |  |  |
| 2 | Age | 42.33 | 10.57 | .12^*^ | - |  |  |  |  |  |  |  |  |  |  |  |  |  |  |  |  |  |  |  |  |
| 3 | Children^b^ | 0.30 | 0.46 | .08 | -.02 | - |  |  |  |  |  |  |  |  |  |  |  |  |  |  |  |  |  |  |  |
| 4 | Working time^c^ | 37.62 | 8.45 | .23^**^ | -.01 | -.21^**^ | - |  |  |  |  |  |  |  |  |  |  |  |  |  |  |  |  |  |  |
| 5 | Autonomy _(b)_ | 3.06 | 0.67 | .07 | .00 | -.02 | .05 | - |  |  |  |  |  |  |  |  |  |  |  |  |  |  |  |  |  |
| 6 | Autonomy _(h)_ | 3.16 | 0.68 | .05 | .00 | -.02 | .03 | .78^**^ | - |  |  |  |  |  |  |  |  |  |  |  |  |  |  |  |  |
| 7 | Overwork^adv^ _(b)_ | 2.02 | 0.78 | .05 | .00 | -.02 | .32^**^ | .07 | .07 | - |  |  |  |  |  |  |  |  |  |  |  |  |  |  |  |
| 8 | Overwork^adv^ _(h)_ | 1.93 | 0.84 | .03 | -.04 | .06 | .26^**^ | -.02 | .02 | .75^**^ | - |  |  |  |  |  |  |  |  |  |  |  |  |  |  |
| 9 | Interruptions^ad^ _(b)_ | 3.05 | 0.79 | -.09 | .08 | -.04 | .22^**^ | .07 | .09 | .45^**^ | .39^**^ | - |  |  |  |  |  |  |  |  |  |  |  |  |  |
| 10 | Interruptions^ad^ _(h)_ | 2.22 | 0.78 | .02 | -.09 | .27^**^ | .10 | .13^*^ | .10 | .30^**^ | .44^**^ | .49^**^ | - |  |  |  |  |  |  |  |  |  |  |  |  |
| 11 | Lack of com-munication _(b)_ | 1.18 | 0.40 | .03 | .05 | -.07 | .09 | .06 | .05 | .03 | .09 | -.07 | .02 | - |  |  |  |  |  |  |  |  |  |  |  |
| 12 | Lack of com-munication _(h)_ | 1.88 | 0.88 | .09 | .10 | -.06 | .05 | .15^**^ | .14^*^ | .07 | -.02 | - .03 | -.19^**^ | .27^**^ | - |  |  |  |  |  |  |  |  |  |  |
| 13 | Work environ-ment^dis^ _(b)_ | 3.10 | 0.60 | -.07 | .08 | .05 | -.02 | .22^**^ | .13^*^ | -.14^*^ | -.11^*^ | -.21^**^ | .09 | .04 | -.08 | - |  |  |  |  |  |  |  |  |  |
| 14 | Work environ-ment^dis^ _(h)_ | 2.70 | 0.72 | .08 | .12^*^ | -.20^**^ | .12^*^ | .04 | .07 | .00 | -.08 | .00 | -.11^*^ | -.02 | -.13^*^ | .19^**^ | - |  |  |  |  |  |  |  |  |
| 15 | Information deficit _(b)_ | 2.41 | 0.77 | .08 | .06 | .05 | .13^*^ | .05 | .07 | .15^*^ | .18^**^ | .28^**^ | .22^**^ | -.03 | .05 | -.19^**^ | -.06 | - |  |  |  |  |  |  |  |
| 16 | Information deficit _(h)_ | 2.80 | 0.79 | .01 | -.03 | .04 | .11 | .03 | .04 | .17^**^ | .23^**^ | .31^**^ | .20^**^ | .02 | .15^**^ | -.14^*^ | -.21^**^ | .73^**^ | - |  |  |  |  |  |  |
| 17 | Job crafting_csr (b)_ | 4.18 | 0.69 | .00 | .12^*^ | .05 | -.05 | .33^**^ | .30^**^ | .08 | .12^*^ | .15^**^ | .17^**^ | -.03 | -.06 | .21^**^ | .12^*^ | - .04 | -.01 | - |  |  |  |  |  |
| 18 | Job crafting_csr (h)_ | 4.11 | 0.77 | .04 | .08 | .08 | -.01 | .29^**^ | .30^**^ | .09 | .18^**^ | .21^**^ | .18^**^ | -.03 | -.11 | .11 | .21^**^ | .01 | -.02 | .78^**^ | - |  |  |  |  |
| 19 | Job crafting_csor (b)_ | 3.12 | 0.92 | .09 | -.19^**^ | .10 | .06 | .10 | .06 | .09 | .15^*^ | .11 | .16^**^ | -.15^**^ | -.07 | .10 | -.05 | - .03 | .06 | .26^**^ | .23^**^ | - |  |  |  |
| 20 | Job crafting_csor (h)_ | 2.77 | 0.94 | .05 | -.15^*^ | .12^*^ | .06 | .06 | -.01 | .00 | .12^*^ | .11 | .21^**^ | -.10 | -.22^**^ | .09 | .06 | .00 | .01 | .26^**^ | .28^**^ | .83^**^ | - |  |  |
| 21 | Emotional exhaustion | 2.15 | 1.00 | -.02 | -.10 | .08 | .04 | -.10 | -.15^*^ | .20^**^ | .24^**^ | .18^**^ | .17^**^ | .10 | -.04 | -.19^**^ | -.26^**^ | .15^**^ | .26^**^ | -.22^**^ | -.29^**^ | -.06 | -.12^*^ | - |  |
| 22 | Work engagement | 3.26 | 0.96 | .05 | .13^*^ | .11 | .10 | .19^**^ | .17^**^ | .10 | .15^**^ | .05 | .06 | .01 | -.08 | .12^*^ | .16^**^ | -.10 | -.15^**^ | .48^**^ | .44^**^ | .26^**^ | .31^**^ | -.32^**^ | - |

*Note.* N = 308 based on listwise deletion; b = job setting before the pandemic; h = homeoffice; csr = increasing structural resources; csor = increasing social resources.^a^ 0 = female, 1 = male. ^b^ 0 = no, 1 = yes. ^c^ working time is measured in hours (hrs); ^ad^ = worded as advantage but coded reversely in the FGBU. ^dis^ = worded as disadvantage but coded reversely in the FGBU.*p < .05. **p < .01.
